# Hey Teacher, Don’t Leave Them Kids Alone: Action Is Better for Memory than Reading

**DOI:** 10.3389/fpsyg.2017.00325

**Published:** 2017-03-09

**Authors:** Mathieu Hainselin, Laurence Picard, Patrick Manolli, Sophie Vankerkore-Candas, Béatrice Bourdin

**Affiliations:** ^1^CRP–CPO, EA 7273, Université de Picardie Jules VerneAmiens, France; ^2^Laboratoire de Psychologie, EA 3188, Université Bourgogne Franche-ComtéBesançon, France

**Keywords:** enactment, memory, action, teaching, education

## Abstract

There is no consensus on how the enactment effect (EE), although it is robust, enhances memory. Researchers are currently investigating the cognitive processes underlying this effect, mostly during adulthood; the link between EE and crucial function identified in adulthood such as episodic memory and binding process remains elusive. Therefore, this study aims to verify the existence of EE in 6–10 years old and assess cognitive functions potentially linked to this effect in order to shed light on the mechanisms underlying the EE during childhood. Thirty-five children (15 second graders and 20 fifth graders) were included in this study. They encoded 24 action phrases from a protocol adapted from Hainselin et al. (2014). Encoding occurred under four conditions: Verbal Task, Listening Task, Experimenter-Performed Task, and Subject-Performed Task. Memory performance was assessed for free and cued recall, as well as source memory abilities. ANOVAS were conducted to explore age-related effects on the different scores according to encoding conditions. Correlations between EE scores (Subject-Performed Task/Listening Task) and binding memory scores (short-term binding and episodic memory) were run. Both groups benefited from EE. However, in both groups, performance did not significantly differ between Subject-Performed Task and Experimenter-Performed Task. A positive correlation was found between EE and episodic memory score for second graders and a moderate negative correlation was found between EE and binding scores for fifth graders. Our results confirm the existence of EE in 6 and 10 year olds, but they do not support the multimodal theory (Engelkamp, 2001) or the “glue” theory (Kormi-Nouri and Nilsson, 2001). This suggests instead that episodic memory might not underlie EE during early childhood.

## Introduction

Memory for action is one way to assess embodied cognition. Within this field, enactment effect (EE) refers to better memory for performed actions than for verbally encoded action sentences. Even if it is a robust effect in adults, there is still no consensus on the conditions revealing this effect in childhood and on the mechanisms enabled its expression. In fact, we still do not know how it enhances memory, and if the same processes are involved in childhood and adulthoo (Madan and Singhal, 2012; Hainselin, 2015). The assessment of the EE is generally done through an action phrase memory task (e.g., “put the glass on the table,” “move the scissors to the left”) when participants perform the actions themselves (Self-Performed Tasks, SPT), watch the actions performed by the experimenter (Experimenter-Performed Tasks, EPT) or only read the sentence (Verbal Tasks, VT). Most experiments include free and/or cued recall, and recognition.

Different theories attempt to explain EE. The multimodal theory (Engelkamp, 2001) supposes that performing an action involve additional motor components in comparison to observing or reading action sentences. Beyond action planning, visual information and movement control, SPT provide a kinaesthetic feedback during information encoding. This theory is not just about deeper processing but multimodality. Engelkamp (2001) emphasized the superiority of EPT or imagining the action compared to VT was not as large as the SPT superiority over VT, in reference to previous findings of better performance in SPT compared to EPT to support this hypothesis (Engelkamp and Zimmer, 1989; Engelkamp, 1995). In a previous study with transient global amnesia patients, we found an improvement of performance between EPT and VT but no difference between SPT and EPT; so it does not fully support the multimodal theory (Hainselin et al., 2014). Feyereisen (2009) found a similar pattern of result (similar benefit of SPT and EPT to VT) in aging. Another theory, named the “glue” theory, stress the importance the core role of cognitive binding of action and object (Kormi-Nouri and Nilsson, 2001). Accordingly, an active learning episode through enactment enhances episodic integration as it strengthens the link between action verbs and nouns. Kormi-Nouri and Nilsson (2001) claims that the two components (action verbs and noun) are encoded in one memory unit or in two close and binded units. For example, if you scratch the brush, the action (scratch) and the object (the brush), it is engrammed in the same episode. The authors assumed that the interaction between self and object is crucial for action memory performance. Few studies conducted with adults argue for this approach, as they showed that the size of the EE is importantly linked with short-term binding abilities (Mangels and Heinberg, 2006; Hainselin et al., 2014). Generally, binding is defined as an associative process that allows integrating features of episodes into coherent representation. Binding could concern information from different sources, both within and between modalities. For example, Baddeley (2000) assumes that the episodic buffer maintain short-term representations using a multidimensional code that notably serve to integrate information from phonological and visuospatial performance. Adopting such a perspective, Hainselin et al. (2014) tested short-term binding abilities (a consonant had to be associated with specific locations in a grid) and looked for their links with the size of the EE in adults. They show that binding performances strongly correlated with EE, arguing in favor of the “Glue theory”.

In the last years, embodied cognition has been a trending topic, showing how cognition is influenced by sensory-motor processing (Barsalou et al., 2003). For example, participants studying angular momentum by physically tilting a set of wheels had better quiz scores than participants only watching it (Kontra et al., 2015). Surprisingly, few studies have investigated EE in children, despite its potential implication for developmental psychology and education. Describing the encoding conditions that are the most appropriate during childhood is in fact essential, especially as large age-related differences in episodic memory abilities have been observed during childhood and adolescence (e.g., Picard et al., 2017). Globally, performing action has been found to improve memory, in normal children (Blake et al., 1987; Ratner and Hill, 1991; James and Swain, 2011; Badinlou et al., 2015), in children with learning disabilities (Freides and Messina, 1986), in children with attention deficit hyperactivity disorder (Yang et al., 2017) or in developmental pathologies, such as autism (for a review, see Grainger et al., 2014). Memorization of sentences seems in fact facilitated when children perform gestures which are coherent with the meaning of sentences in comparison to verbal learning, that is learning by reading or listening an action sentence. How this pattern varies according to age is, however, a still debated question. Some studies showed in fact that no developmental difference in performance is observed after an action encoding condition (e.g., Cohen and Stewart, 1982) whereas others observed that age-related differences in recall scores remain (e.g., Kormi-Nouri et al., 2008). Contradictory results also concern the effect of the interaction between self and objects. Thus, in agreement with the multimodal theory, some studies observed an advantage of SPTs over EPT in children (e.g., Baker-Ward et al., 1990), whereas others reveal the opposite pattern (Ratner and Hill, 1991) or comparable performance (e.g., Foley and Johnson, 1985). In order to go over the methodological gap of previous studies, an EE paradigm has been recently proposed to children from 8 to 14 years, using the three most common encoding conditions: VT, EPT, and SPT (i.e., Read, Watch, and Perform; Badinlou et al., 2015) and three retrieval phases: free recall, cued recall, and then recognition tests. This research revealed several differences between SPTs and EPTs in their school-aged participants. During free and cued recall, age-related increase in performance was in fact more important for SPTs than for EPTs or VT, which did not differ. Moreover, the developmental effect persisted during recognition for SPT, while it disappeared for EPT (and VT). These results have to be replicated, and completed with another encoding condition, particularly important from an educational perspective: the Listening Task. Listening is probably, with Watching, the most common encoding condition at school and should thus be compared with the three other conditions.

The reasons behind the EE remain also unclear from a developmental perspective. Which are the cognitive processes that underlie this effect and its potential development? Recently, some studies have sought to identify factors that could explain EE variance in childhood, among which verb type, time of testing (immediate or delay), or language proficiency (Frick-Horbury, 2002; de Nooijer et al., 2013, 2014). To our knowledge, no study has yet addressed the role of cognitive processes (binding, episodic memory) possibly implicated in the EE from a developmental perspective, although theoretical frameworks underlined these cognitive processes’ involvement. It is especially important to assess their possible contribution that both short- and long-term binding abilities have been shown to increase largely throughout childhood (Lee et al., 2016; Picard et al., 2017). Thus, this study aimed to (1) verify the existence of EE in 6–11 years old children using four different encoding conditions (VT, LT, EPT, SPT), and (2) assess the link between EE and short-term binding and episodic memory, in order to improve general knowledge about EE and its mechanisms during childhood.

## Materials and Methods

### Participants

Participants were 15 second graders (9 girls and 6 boys) and 20 fifth graders (9 girls and 11 boys) from different elementary schools in France. They were all native French speakers. For the first graders group, the mean age was 7.6 (*SD* = 1.9, range: 7.3–7.9 years) and for the fifth graders, the mean age was 10.6 (*SD* = 5.7, range: 9.8–11.3 years). Experiments were carried out in calm offices in the children’s schools. The children’s participation was conditioned by their parents’, headmasters’, and teachers’ approval, and their own willingness to take part in the experiment. The children’s parents filled in a questionnaire to ensure the absence of a background of neurological or psychiatric medical history, developmental learning disorders and repetition of a grade at school, which constituted exclusion criteria. Participants also had to have normal fluid reasoning performance. To assess it, the Matrix Reasoning subtest of the WISC-IV (Wechsler, 2005) was proposed. Children were presented with a partially filled grid and asked to select one out of four or six items that properly completes the matrix considering a logical criterion. One point was given for each correct answer and testing stop when participants failed to answer correctly to four among the last five items. Standard score had to be in the normal range (4–16); based on this criterion, one boy from the fifth graders group was excluded from the analysis. In the end, we had 19 fifth graders (9 girls and 10 boys). This study was carried out in accordance with the recommendations of French law written informed consent from all subjects. All participants gave written informed consent in accordance with the Declaration of Helsinki.

### Materials

Twenty-four poorly integrated action phrases (one action verb + one object noun) were used for the enactment task. Action phrases were created using a list of 24 actions and 24 objects inspired from Hainselin et al. (2014). Action-object pairing was randomly determined. However, associations were controlled so that a possible link exists between the action and the object. Consequently, associations such as “fold the torch in half” were excluded. Actions were chosen to be low associates of the objects with which they were paired and vice versa (i.e., prototypical association such as “read a book” were excluded to avoid answers based on semantic knowledge and to reinforce involvement of binding processes). The enactment task was designed on E-Prime 2.0 and was presented using a 15.4-inch screen ASUS laptop. Participants were seated 50–60 cm away from the computer screen. For the complementary cognitive assessment tasks, verbal memory was assessed with the Narrative Memory subtest (NEPSY-II, Korkman et al., 2012) whereas short-term binding capacity was assessed with a binding task inspired from Picard et al. (2012) and run with E-Prime 2.0.

### Procedure

Children were tested individually in a separate room in their schools by an experimenter. Each session lasted about an hour. Children first performed the Enactment task after which they completed the Matrix Reasoning subtest (Wechsler, 2005), that also served as interference task. After the Enactment retrieval phases, subjects successively performed the short-term binding task and the Narrative Memory task (Korkman et al., 2012). Tests were administered in the same order for all children. Moreover, children were presented with examples and training for all tasks in order to ensure that all instructions have been fully understood.

#### Enactment Task

**FIGURE 1:**
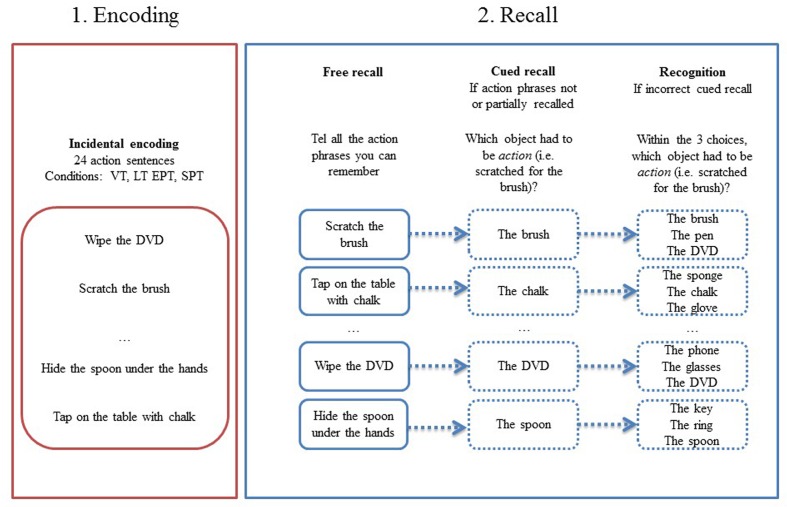
**Design for the enactment effect task**. After encoding 24 actions, participants have to retrieve actions in free and cued recall. There was a recognition for items incorrectly recalled in the free or cued recall. VT, Verbal Task; LT, Listening Task; EPT, Experimenter-Performed Task; SPT, Self-Performed Task.

Participants incidentally encoded 24 poorly integrated action phrases under four conditions: Read out loud (VT), Listen (LT), Watch (EPT), and Perform (Subject-Performed task, SPT). We took poorly integrated (scratch the rub) instead of typical (erase with the rub) action phrases (well integrated) because we wanted to assess the glue theory. With typical action phrases, participants would not had to bind the action and object together as it is already well integrated (Mangels and Heinberg, 2006). In addition, when using typical action phrases, it is very difficult to know if participants genuinely remember the action associated to the object or if they give the most obvious one; for example: erase is generally the first word you can think about after seeing a rubber. Six action phrases were presented for each encoding condition with a fixed order for all subjects. Each action phrase was displayed on the screen for 5 s during which the subject had to read it silently. Once the action phrase appeared, the object mentioned in the action phrase was physically presented in front of the child. Then the encoding condition appeared above the action phrase and children had to perform the indicated action. For the VT, the participants were instructed to read out loud the phrase; for the LT, they had to listen to the experimenter that read the sentence; for the EPT, they were instructed to watch the experimenter performing the action with the present object; for the SPT, they had to carry out the indicated action with the object in front of them. Once the indicated action has been performed, object was removed and the following action phrase appeared. Action-object pairing and encoding conditions were counterbalanced in two lists to avoid item order bias. Before beginning, children were provided with one example for each condition to ensure that instructions have been fully understood (see **Figure 1** for design).

After performing the interference task (Matrix Reasoning subtest, Wechsler, 2005) which lasted around 15 min, children performed three successive memory tests: a free recall, a cued recall, and a recognition test. In the free recall test, they were instructed to recall a maximum number of associations (action + object). If associations cannot be retrieved, they were asked to recall action and objects separately. For each action phrase correctly recalled, participants performed a source memory task, as they had to indicate the encoding condition (i.e., Read, Listen, Watch, Perform).

For action phrases that were not or only partially recalled, participants performed a cued recall task: the action was provided as a cue to the object from the encoded action phrase (e.g., what were you required to “fold in half”?). When the cued recall task did not allow retrieving the correct object, a forced choice recognition task was proposed: children had to detect the object associated with the action among a distractor from the same encoding condition as the target and another from a different encoding condition (e.g., “were you required to touch your nose with the modeling clay, with the plastic glove, or with the paintbrush?”). All the proposed items have been previously encoded in order to avoid answers based only on familiarity processes. Position of the cue among the three propositions varied: for one third of the trials the cue object was proposed first, for one third of the trial was proposed in the middle position, and for the last third of the trials it was in the last position.

We marked one point for each correctly retrieved information. Thus, in the free recall task, two points were awarded for each action phrase completely recalled (verb + object), and a single point for each partial recall (i.e., only object or action). The maximum Free Recall score is then 12 for each condition, and 48 for the Total Free Recall score (the sum of the free recall scores for all four conditions). Similarly, one point was awarded for correct answers in cued recall and recognition. As cued retrieval was not proposed for associations correctly remembered during free recall, cued recall scores correspond to the sum of free and cued recall answers. The maximum score was then of 12 for each conditions, and 48 for the Total Cued Recall score. For a better understanding, all scores were converted into percentages. Similarly, the maximum recognition score was of 12 for each condition, and 48 for the Total Recognition score. Source score was calculated as percentage taking correct answer into account.

In order to have an indicator of the size of the EE, the *classic improvement index* (Perform/Read) was calculated by dividing the Recognition score in the Perform condition by the Recognition score in the Read condition (for similar method, see Hainselin et al., 2014). Given that the youngest participants are only 7 years old in average, reading abilities are not fully automatized. This lack of automaticity could put the youngest’ enactment index on a disadvantage. Thus, in an exploratory perspective, we also calculated a second EE index, the *Listen improvement index*, based on the Listen, instead of the Read condition. It was calculated by dividing the Recognition score in the SPT (Perform) condition by the Recognition score in the VT (Listen) condition.

#### Cognitive Assessment

##### Short-term feature-binding

**FIGURE 2:**
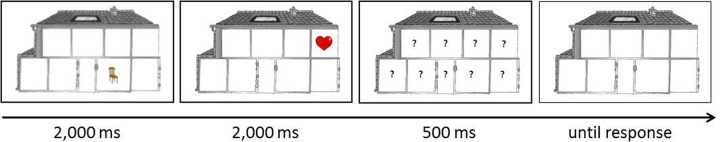
**Example of a trial (*N = 2*) taken from the short-term feature-binding task adapted from Picard et al. (2012)**.

The task designed to assess the capacity to bind various features during short delay was inspired by the original task created by Prabhakaran et al. (2000) and adapted for children in a non-verbal fashion (e.g., Lorsbach and Reimer, 2005). The task required to memorize during short delay associations between verbal, spatial, and temporal information (for a very close task, see Picard et al., 2012). In the task used here, children had to memorize strings of objects (2, 3, or 4 objects) that appear one after another in a specific spatial context (the rooms of a house display; **Figure 2**), in order to recall the three bounded features straight after: verbal feature (name of the object), spatial feature (location), and temporal information (recall had to be in the same order as the presentation). Nine colorized line drawings of common objects were used. All of them were one syllable long, were not complex (Mean = 2.5/5), familiar (Mean = 2.5/5), and very frequently correctly named (Mean = 90.5%) according to the norms published by Rossion and Pourtois (2004). Stimuli were displayed using E-Prime 2.0: objects were displayed for 2000 ms with an inter-stimulus interval of 500 ms. The end of the trial was symbolized by question marks that remained on the screen for 500 ms. The participant then had to orally recall the sequence of encoded objects, pointing to their respective locations, in their order of appearance. The diversity of the representation that have to be associate was strengthen by the use of an oral recall task instead of a recognition one, as it encourage children to maintain object information according to a phonological code rather than a visual ones. The task consisted of two examples and nine trials (three trials for each length), which were presented randomly but in the same order for all participants. One point was awarded for each sequence of items correctly recalled regardless of its length. The maximum score is then equal to nine.

##### Verbal episodic memory

The Narrative Memory subtest of the NEPSY-II (Korkman et al., 2012) was used to assess memory for organized verbal material under free and cued recall. The children listened to a brief story and were then asked to recall its main points. In the free recall phase, one point was given for each of the 20 expected details spontaneously recalled (Free Recall score, max = 20). For each missing detail, a cued recall task was proposed, in the form of a question (e.g., “what was the name of the child?”). One point was given for each additional detail recalled. The Cued Recall score consisted of the sum of the freely recalled details (two point for each detail), and the cued recalled details (one point for each detail; max = 40). We used the Cued Recall score, taking the Free Recall score into account, in our analysis.

### Statistical Methodology

The statistical analysis of the enactment task scores was carried out using repeated measures ANOVAs, with Group (Second Grader, Fifth Grader) as the between participants factor, and Condition (Read, Listen, Watch, Perform) as the within-participants factor, in order to assess the effect of age groups and encoding conditions. The short-term binding and the episodic memory scores were analyzed using a one-way ANOVA, with Group as a between-participants factor. *Post hoc* Tukey tests were used to carry out paired comparisons. Finally, in order to determine which processes were linked to the EE, Pearson’s correlation coefficients were calculated between cognitive scores and the EE (using the *classic improvement index* Perform/Read and the *Listen improvement index* Perform/Listen). Analyses were conducted successively for each age group, to detect potential differences in mechanisms associated with age. All analyses were performed with JASP Team (2016).

## Results

### Enactment Task

**FIGURE 3:**
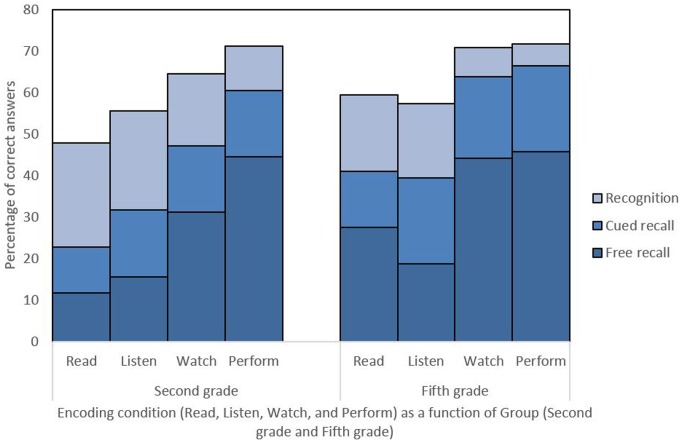
**Percentage of correct answers of action events recalled as a function of type of encoding condition (Read, Listen, Watch, Perform), Group (Second Graders and Fifth Graders), and Retrieval phase (Free Recall, Cued Recall, Recognition)**.

Descriptive data are presented in **Figure 3** and raw data are available in Supplementary Data Sheet 1.

### Free Recall

A main effect of Group was found [*F*(1,32) = 7.50, *p* = 0.01, η^2^ = 0.19], such that Fifth Graders recalled more items than Second Graders. An effect of encoding condition was also found [*F*(3,32) = 24.81, *p* < 0.001, η^2^ = 0.43], such that the performance in the Watch and Perform conditions were better than that of the Read and Listen conditions (*p* < 0.001 for all significant comparisons). The Group × Condition interaction was not significant [*F*(3,32) = 1.48, *p* = 0.23, η^2^ = 0.03]. See Supplementary Data Sheet 2 for detailed recall scores.

### Cued Recall

The ANOVA for the cued recall memory performance revealed a very similar pattern: a main effect of Group [*F*(1,32) = 14.00, *p* < 0.001, η^2^ = 0.30], with Fifth Graders recalling more items than Second Graders, an effect of encoding Condition [*F*(3,32) = 40.96, *p* < 0.001, η^2^ = 0.55], with Watch and Perform items better recalled than that of the Read and Listen conditions (*p* < 0.001 for all significant comparisons), and no Group × Condition interaction [*F*(3,32) = 1.47, *p* = 0.23, η^2^ = 0.02].

### Recognition

The ANOVA conducted on the recognition scores showed again a main effect of Group [*F*(1,32) = 6.98, *p* = 0.01, η^2^ = 0.18], such that Fifth Graders recalled more items than Second Graders, an effect of Condition [*F*(3,32) = 25.05, *p* < 0.001, η^2^ = 0.43], such that the performance in the Watch and Perform conditions were better than that of the Read and Listen conditions, and no Group × Condition interaction [*F*(3,32) = 1.82, *p* = 0.15, η^2^ = 0.03].

### Enactment Indexes

The ANOVAs conducted on the classic enactment index (Perform/Read) did not show a Group effect [*F*(1,32) = 1.60, *p* = 0.22, η^2^ = 0.05], while there was a Group effect for the second enactment index (Perform/Listen), [*F*(1,32) = 11.73, *p* < 0.01, η^2^ = 0.27).

### Source

The ANOVA conducted on the source score showed a main effect of Group [*F*(3,32) = 4.84, *p* = 0.04, η^2^ = 0.14], such that Fifth Graders recalled the encoding condition better than Second Graders, an effect of Condition [*F*(3,32) = 45.77, *p* < 0.001, η^2^ = 0.58], such that the performance in the Watch and Perform conditions were better than that of the Read and Listen conditions, and no Group × Condition interaction [*F*(3,32) = 2.11, *p* = 0.10, η^2^ = 0.03].

### Cognitive Assessment

There is a statistical tendency for a difference between the two groups, with better performance for Fifth Graders than Second Graders for Short-Term Binding [*F*(1,32) = 3.83, *p* = 0.06, η^2^ = 0.11], and Narrative Recall performance [*F*(1,32) = 3.87, *p* = 0.06, η^2^ = 0.11] (see **Table 1**).

**Table 1:** Mean total scores (standard deviation) of the Second and Fifth Graders for the complementary cognitive assessment battery and ANOVAs results.

	Second Graders (*n* = 15)	Fifth Graders (*n* = 19)	Age effect *F*(1,32)
Binding score	5.93 (1.22)	6.89 (1.52)	3.83^†^
Narrative Recall score	19.00 (5.15)	24.00 (8.46)	3.87^†^

*^†^*p* = 0.06*.

### Correlation

For all participants and for Fifth Graders, we found a negative correlation between the Enactment Index and the binding score (respectively, *r* = -0.40 and *r* = -0.49; i.e., the fewer correct answers provided by children in the binding task, the more their memory was enhanced in the Perform condition in comparison with the Read condition). For Second Graders, a positive correlation was found between the Enactment Index and Narrative Recall (*r* = 0.81) scores. There was no significant correlation between the second index (Perform/Listen) and binding or narrative recall scores (see **Table 2**). See Supplementary Data Sheet 3 for detailed correlation scores.

**Table 2:** Pearson’s correlation between Enactment Indexes and cognitive scores for Second Graders and Fifth Graders.

		Second Graders (*n* = 15)	Fifth Graders (*n* = 19)
Perform/Read (classic index)	Binding score	-0.10	-**0.49**^∗^
	Narrative Recall score	**0.81**^∗∗∗^	-0.38
Perform/Listen (Listen index)	Binding score	0.01	-0.35
	Narrative Recall score	0.42	-0.13

*Significant correlations are in bold*.

*^∗^*p* < 0.05*.

*^∗∗∗^*p* < 0.001*.

## Discussion

### Enactment Theories

Our results confirmed the presence of an EE in children and the benefit of performing actions for learning purposes. Although we confirmed previous results on the importance of performing actions to enhance memory through the EE, our results are not consistent with the multimodal theory: Watch and Perform conditions performance did not differ for free and cued recalls nor recognition.

Several studies reported that sentences were remembered better if the children mimed the content of the sentences rather than if they were verbally rehearsed (Saltz and Dixon, 1982; Blake et al., 1987; Ratner and Hill, 1991; James and Swain, 2011). Our results showed also that children, whatever their age, exhibited higher recall performance when they had to perform the actions indicated in the sentences than when they have to listen. They also indicate that EE is observed in the subject-performed condition (differences between Perform and Read) and, inconsistently with Badinlou et al.’s (2015) results, in the experimenter-performed condition (differences between Watch and Read) This might be due to our sample size or the cued recall methodology, for which we only ask for items participants did not recalled freely, contrary to Badinlou et al. (2015) who asked participants to recall every item during cued recall, including those recalled during free recall. With Badinlou et al.’s (2015) methodology, participants with correct answers on free recall could fail to give the correct answer during cued recall, whereas this is not possible with our methodology.

The better performance in memory for action conditions (Watch and Perform) over non-active conditions is also highlighted for source memory (i.e., encoding condition). This adds evidence to the superiority of action conditions, not only for items association, as previously described in the literature, but also for its context. Taken together these results suggest that physical activity, by children themselves, although preferable, is not indispensable to the EE in younger children. Younger children (Second Graders) recall as much as older children (Fifth Graders) when they learn through action but not when they watch the experimenter performing the actions. Although younger children have lower memory performance than older children, this difference disappears when they learn through action, which is the best encoding condition for both groups. These results are not reflecting a ceiling effect, as our participants have an 80% success rate for SPT. The importance of actions in cognitive development is known in the education of young children (Rubin et al., 1983; Cohen, 1989), with better learning while acting (Ballantyne and Packer, 2009). While the Listening condition is not always assessed, children exhibited worse memory performance under it for all retrieval conditions, compared to EPT or SPT. Therefore, under this condition, teachers, clinicians, and scientists might under-estimate children’s capacities of memorization. In the future, there is a need for more research on memory for action development, with a specific focus on the cognitive processes linked to the EE, such as working memory, which is very important in episodic memory development (Picard et al., 2009; Rhodes et al., 2011; Rajan et al., 2014).

### Pedagogical Issues

It has been shown that action enactment facilitates verbal memory for children. Our results support this finding and research results should encourage teachers to support a more active learning, in particular in young children. Previous publications has shown that watching or performing gestures facilitates acquisition of a variety of tasks, such as mathematical tasks (Cook et al., 2008; Novack et al., 2014) and vocabulary learning (Macedonia and Klimesch, 2014; Mavilidi et al., 2015; Toumpaniari et al., 2015). In particular, foreign language vocabulary learning is easier in preschool children (4–5 years old) when congruent gestures are used in word learning (Mavilidi et al., 2015; Toumpaniari et al., 2015). Gestures can also foster text learning (Cutica et al., 2014).

We did not confirm the glue theory either, as we did not find positive correlation between enactment index score and binding performance (for both enactment indexes). This was the first time that the link between binding and EE was assessed during childhood. As expected, short-term binding performance increases with age, in agreement with previous works (e.g., Gray et al., 2017), confirming that our task was appropriate. When using the classical index (Perform/Read), we did not observe any link with binding for Second Graders and found a negative correlation for the Fifth Graders Group. This unexpected result suggests that the EE might not be supported by the same cognitive processes in childhood as in adulthood. In line with previous work that suggest a need of a different theoretical framework for EE in amnestic patients (Hainselin et al., 2014), it seems that our results cannot be completely explained by the glue theory. In this perspective, research with amnesic children should lighten this actual gray area. Future works should deepened the link between EE and binding process. As only one specific short-term binding task was used here whereas several types of binding could be identified (see for example Gray et al., 2017). More studies are needed to test if the actual results are also observed with other kind of associative processes. Besides, we tried to use here a specific index to avoid any reading ability bias that could lead to differences between age groups. The sensitivity of this new index was not clearly observed here and identification of a new and more appropriate index to reveal EE should pursuit.

The majority of activities in school mostly are based on verbal tasks. Therefore, it is possible that memorization capacity is under-estimated in young children who have a less-developed language capacity or in learning disabled children. In accordance with this conception, a study has shown that children with autism spectrum disorders have better performance when they acted than in another watching condition (Lind and Bowler, 2009). In the same way, learning disabled children, known to have difficulties to memorize verbal information, have memory scores equivalent to the normal control in the motor enactment condition (Freides and Messina, 1986). In our research, children with lower performance can benefit from actions, and future long term research is needed to confirm whether this is an efficient method to improve teaching and reduce inequality between children. In conclusion, we encourage teachers to bring more actions to the classroom, and scientists to evaluate the EE for children with learning disabilities.

## Author Contributions

MH: Conception and design of the task, analysis and interpretation of the data, writing the manuscript. LP: Conception and design of the task, analysis and interpretation of the data, writing the manuscript. PM: Acquisition of the data, drafting the manuscript. SV-C: Acquisition of the data, drafting the manuscript. BB: Conception and design of the task, analysis and interpretation of the data, writing the manuscript.

## Conflict of Interest Statement

The authors declare that the research was conducted in the absence of any commercial or financial relationships that could be construed as a potential conflict of interest.
