# Supplementary material for: Hey Teacher, Don’t Leave Them Kids Alone: Action Is Better for Memory than Reading
Source: Front Psychol. 2017 Mar 9;8:325. doi: 10.3389/fpsyg.2017.00325 (PMC5343022; doi:10.3389/fpsyg.2017.00325)
Supplement: Supplementary file 2 [file Data_Sheet_2.docx]

Appendix

*Appendix*. Mean total scores for second and fifth graders as a function of encoding (Read, Listen, Watch, Perform) and retrieval condition (Free recall, Cued recall, Recognition) (SD: Standard Deviation)

|  |  | Second Graders | SD | Fifth Graders | SD |
| --- | --- | --- | --- | --- | --- |
| **Free Recall** | *Read* | 1.40 | 1.30 | 3.37 | 2.37 |
|  | *Listen* | 1.87 | 1.85 | 2.37 | 1.84 |
|  | *Watch* | 3.73 | 2.63 | 5.37 | 2.34 |
|  | *Perform* | 5.33 | 2.23 | 5.68 | 1.03 |
| **Cued Recall** | *Read* | 2.73 | 1.71 | 5.00 | 2.32 |
|  | *Listen* | 3.80 | 2.08 | 4.84 | 1.87 |
|  | *Watch* | 5.67 | 2.29 | 7.74 | 1.71 |
|  | *Perform* | 7.27 | 1.75 | 8.16 | 0.93 |
| **Recognition** | *Read* | 5.73 | 1.33 | 7.21 | 1.82 |
|  | *Listen* | 6.67 | 1.11 | 7.00 | 1.03 |
|  | *Watch* | 7.73 | 1.58 | 8.58 | 1.23 |
|  | *Perform* | 8.53 | 1.41 | 8.79 | 0.52 |
