# Supplementary material for: Hey Teacher, Don’t Leave Them Kids Alone: Action Is Better for Memory than Reading
Source: Front Psychol. 2017 Mar 9;8:325. doi: 10.3389/fpsyg.2017.00325 (PMC5343022; doi:10.3389/fpsyg.2017.00325)
Supplement: Supplementary file 3 [file Data_Sheet_3.docx]

| **Pearson Correlations (Second Grade)** | | | | | | | | | | | | | |
| --- | --- | --- | --- | --- | --- | --- | --- | --- | --- | --- | --- | --- | --- |
|  | |  | | **Enactment Index** | | **Enactment Listen index** | | **Binding score** | | **Narrative memory CR** | | **Fluid Reasoning** | |
| Enactment Index |  | Pearson's r |  | — |  | 0.651 | ** | -0.098 |  | 0.807 | *** | 0.189 |  |
|  |  | p-value |  | — |  | 0.009 |  | 0.727 |  | < .001 |  | 0.501 |  |
| Enactment Listen index |  | Pearson's r |  |  |  | — |  | 0.004 |  | 0.421 |  | -0.122 |  |
|  |  | p-value |  |  |  | — |  | 0.990 |  | 0.118 |  | 0.664 |  |
| Binding score |  | Pearson's r |  |  |  |  |  | — |  | -0.193 |  | -0.171 |  |
|  |  | p-value |  |  |  |  |  | — |  | 0.492 |  | 0.542 |  |
| Narrative memory CR |  | Pearson's r |  |  |  |  |  |  |  | — |  | 0.130 |  |
|  |  | p-value |  |  |  |  |  |  |  | — |  | 0.644 |  |
| Fluid Reasoning |  | Pearson's r |  |  |  |  |  |  |  |  |  | — |  |
|  |  | p-value |  |  |  |  |  |  |  |  |  | — |  |
|  | | | | | | | | | | | | | |
| * p < .05, ** p < .01, *** p < .001 | | | | | | | | | | | | | |

| **Pearson Correlations (Fifth Grave)** | | | | | | | | | | | | | |
| --- | --- | --- | --- | --- | --- | --- | --- | --- | --- | --- | --- | --- | --- |
|  | |  | | **Enactment Index** | | **Enactment Listen index** | | **Binding score** | | **Narrative memory CR** | | **Fluid Reasoning** | |
| Enactment Index |  | Pearson's r |  | — |  | 0.587 | ** | -0.491 | * | -0.379 |  | -0.313 |  |
|  |  | p-value |  | — |  | 0.008 |  | 0.033 |  | 0.109 |  | 0.193 |  |
| Enactment Listen index |  | Pearson's r |  |  |  | — |  | -0.353 |  | -0.127 |  | -0.490 | * |
|  |  | p-value |  |  |  | — |  | 0.138 |  | 0.605 |  | 0.033 |  |
| Binding score |  | Pearson's r |  |  |  |  |  | — |  | 0.205 |  | 0.317 |  |
|  |  | p-value |  |  |  |  |  | — |  | 0.400 |  | 0.186 |  |
| Narrative memory CR |  | Pearson's r |  |  |  |  |  |  |  | — |  | 0.436 |  |
|  |  | p-value |  |  |  |  |  |  |  | — |  | 0.062 |  |
| Fluid Reasoning |  | Pearson's r |  |  |  |  |  |  |  |  |  | — |  |
|  |  | p-value |  |  |  |  |  |  |  |  |  | — |  |
|  | | | | | | | | | | | | | |
| * p < .05, ** p < .01, *** p < .001 | | | | | | | | | | | | | |
